# Smart Tumor Cell‐Derived DNA Nano‐Tree Assembly for On‐Demand Macrophages Reprogramming

**DOI:** 10.1002/advs.202307188

**Published:** 2023-12-25

**Authors:** Zhiguo Chen, Sha Yang, Zhuyang Zhao, Liu Feng, Jing Sheng, Ruijia Deng, Binpan Wang, Yuan He, Dan Luo, Ming Chen, Lei Chen, Kai Chang

**Affiliations:** ^1^ Department of Clinical Laboratory Medicine, Southwest Hospital Army Medical University (Third Military Medical University) 30 Gaotanyan, Shapingba District Chongqing 400038 China; ^2^ Department of Gastroenterology, Southwest Hospital Army Medical University (Third Military Medical University) 30 Gaotanyan, Shapingba District Chongqing 400038 China; ^3^ Department of Biological and Environmental Engineering Cornell University Ithaca NY 14853‐5701 USA

**Keywords:** bioimaging, CpG‐ODNs, macrophages reprogramming, non‐linear hybridization chain reaction

## Abstract

Without coordinated strategies to balance the population and activity of tumor cells and polarized macrophages, antitumor immunotherapy generally offers limited clinical benefits. Inspired by the “eat me” signal, a smart tumor cell‐derived proximity anchored non‐linear hybridization chain reaction (Panel‐HCR) strategy is established for on‐demand regulation of tumor‐associated macrophages (TAMs). The Panel‐HCR is composed of a recognition‐then‐assembly module and a release‐then‐regulation module. Upon recognizing tumor cells, a DNA nano‐tree is assembled on the tumor cell surface and byproduct strands loaded with CpG oligodeoxynucleotides (CpG‐ODNs) are released depending on the tumor cell concentration. The on‐demand release of CpG‐ODNs can achieve efficient regulation of M2 TAMs into the M1 phenotype. Throughout the recognition‐then‐assembly process, tumor cell‐targeted bioimaging is implemented in single cells, fixed tissues, and living mice. Afterward, the on‐demand release of CpG‐ODNs regulate the transformation of M2 TAMs into the M1 phenotype by stimulating toll‐like receptor 9 to activate the NF‐κB pathway and increasing inflammatory cytokines. This release‐then‐regulation process is verified to induce strong antitumor immune responses both in vitro and in vivo. Altogether, this proposed strategy holds tremendous promise for on‐demand antitumor immunotherapy.

## Graphical abstract

A proximity anchored non‐linear hybridization chain reaction (Panel‐HCR) strategy is developed for recognition of tumor cells and regulation of M2‐type tumor‐associated macrophages (TAMs) into M1 phenotypes. This Panel‐HCR strategy is composed of two modules: a recognition‐then‐assembly module and a release‐then‐regulation module. This proposed strategy holds tremendous promise for on‐demand antitumor immunotherapy.

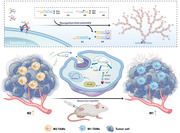

## 1 Introduction

The tumor microenvironment (TME) is characterized by interactions between the immune system and tumor, and it is mainly composed of immune cells, tumor cells, fibroblasts, and extracellular components.^[^ ^1^ ^]^ Tumor‐associated macrophages (TAMs), which account for 50% of the tumor mass and are critical drivers in the formation of an immunosuppressive TME, not only suppress T‐cell activation but also promote tumor metastatic extravasation.^[^ ^2^, ^3^, ^4^ ^]^ TAMs are constantly changing phenotypes of polarized M1‐M2 TAMs under the regulation of tumor cells.^[^ ^5^, ^6^, ^7^ ^]^ M1‐type TAMs have strong anti‐tumor activity, since they kill tumor cells through phagocytosis and also secrete pro‐inflammatory factors to mediate cytotoxicity.^[^ ^8^, ^9^, ^10^ ^]^ In contrast, M2‐type TAMs can promote tumor proliferation, metastasis, and infiltration by the secretion of a variety of tumor‐supporting mediators.^[^ ^11^, ^12^, ^13^ ^]^ These findings make the regulation of TAMs a feasible approach for tumor treatment.^[^ ^14^, ^15^ ^]^ Moreover, the occurrence and metastasis of tumors depend on the presence of viable tumor cells in a supportive microenvironment.^[^ ^16^, ^17^ ^]^ Thus, the recognition of tumor cells and subsequent regulation of M2‐type TAMs into M1 phenotypes are expected to serve as a promising strategy for precise tumor treatment.

DNA nanotechnology is a rapidly developing field that uses the Watson‐Crick base pairing rules of DNA to design artificial nucleic acid structures with specific structures and functions.^[^ ^18^, ^19^, ^20^, ^21^ ^]^ Taking advantage of programmability and recognition properties, DNA nanotechnology is considered as a powerful platform for bioimaging, bioassay, and drug delivery.^[^ ^22^, ^23^, ^24^ ^]^ To precisely identify tumor cells, several approaches have been developed to characterize cell‐specific proteins based on DNA nanotechnology.^[^ ^25^, ^26^, ^27^, ^28^ ^]^ Among these approaches, proximity ligation assay (PLA) is a new strategy in which the target molecules are simultaneously recognized by paired proximity probes.^[^ ^29^, ^30^, ^31^, ^32^ ^]^ The ligation of proximity probes can transform the protein‐recognition signals into DNA detection with the nucleic acid amplification method.^[^ ^33^, ^34^, ^35^, ^36^ ^]^ The unique mechanism of PLA provides many application possibilities in adherent cell lines, cytospin preparations, and tissues.^[^ ^37^ ^]^ However, because of the use of nucleic acid amplification, PLA analysis of cells or tissues requires fixation and prior permeabilization, thus limiting the application of PLA in tumor cell detection.^[^ ^38^ ^]^ Therefore, it is necessary to improve the nucleic acid amplification process of PLA for the recognition of tumor cells and regulation of TAMs.

As an enzyme‐free, sensitive, and rapid analytical technique, non‐linear hybridization chain reaction (HCR) has been applied to visualize low‐expression intracellular RNA.^[^ ^39^, ^40^ ^]^ In the initial non‐linear HCR, a single‐stranded DNA triggers the hybridization‐strand displacement‐dissociation‐rehybridization cycles of four programmed DNA probes, leading to the formation of a DNA nano‐tree containing repetitive segments and the release of byproducts.^[^ ^41^ ^]^ Our group reported an enzyme‐free electrochemical biosensor for ultrasensitive microRNA detection based on non‐linear HCR.^[^ ^40^ ^]^ The non‐linear HCR showed great potential for solving problems in the traditional nucleic acid amplification process of PLA, such as enzyme dependence, non‐isothermality, and complex operation.^[^ ^42^, ^43^, ^44^, ^45^ ^]^ In addition to the nonlinear assembly mechanism, the released byproducts during the DNA nano‐tree growth can be utilized to regulate TAMs. CpG oligodeoxynucleotides (CpG‐ODNs), as toll‐like receptor 9 (TLR9) agonists, have been tested for their ability to regulate TAMs through initiating MyD88‐dependent signaling pathways.^[^ ^46^, ^47^, ^48^, ^49^, ^50^ ^]^ Therefore, the conjugation of CpG‐ODNs to byproducts could be an efficient process for the regulation of M2‐type TAMs into M1 phenotypes in the DNA nano‐tree assembly process.

**Scheme 1:**
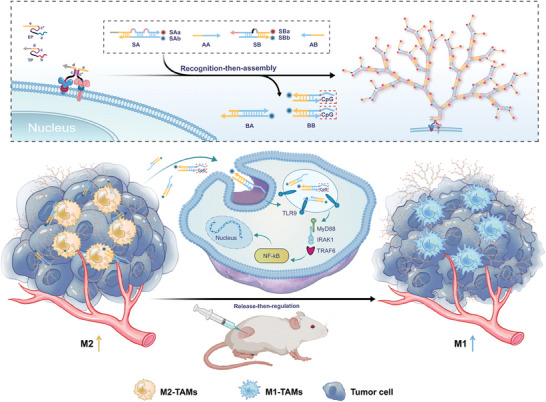
Scheme of smart tumor cell‐derived DNA nano‐tree assembly for on‐demand macrophages reprogramming.

In this study, we developed a proximity anchored non‐linear hybridization chain reaction (Panel‐HCR) strategy for recognition of tumor cells and regulation of M2‐type TAMs into M1 phenotypes. As shown in Scheme 1, this Panel‐HCR is composed of two modules: a recognition‐then‐assembly module and a release‐then‐regulation module. To sense the tumor cell‐specific proteins, TLS11a and epithelial cell adhesion molecule aptamer (EpCAM) with extended proximity (Regions c and c*) and triggering (Regions d and e) sequences were selected as the paired proximity probes. The specific recognition of proximity probes triggered a non‐linear HCR, resulting in the assembly of DNA nano‐tree on the surfaces of tumor cells and the release of CpG‐ODN‐loaded byproduct strands to the tumor microenvironment. Importantly, the release of CpG‐ODNs depended on the concentration of tumor cells. The on‐demand release of CpG‐ODNs could efficiently repolarize M2 TAMs into the M1 phenotype in vivo by changing the interaction with TLR9 and regulating the induction of cytokines. As a proof of concept, we verified the potential of this Panel‐HCR for tumor cell‐derived DNA nano‐tree assembly and release of CpG‐ODNs for TAMs regulation, providing a feasible approach for antitumor immunity and facilitating the biomedical application of DNA nanotechnology.

## 2 Results

### 2.1 Design of Recognition‐Then‐Assembly Module and Release‐Then‐Regulation Module for Panel‐HCR

**Figure 1:**
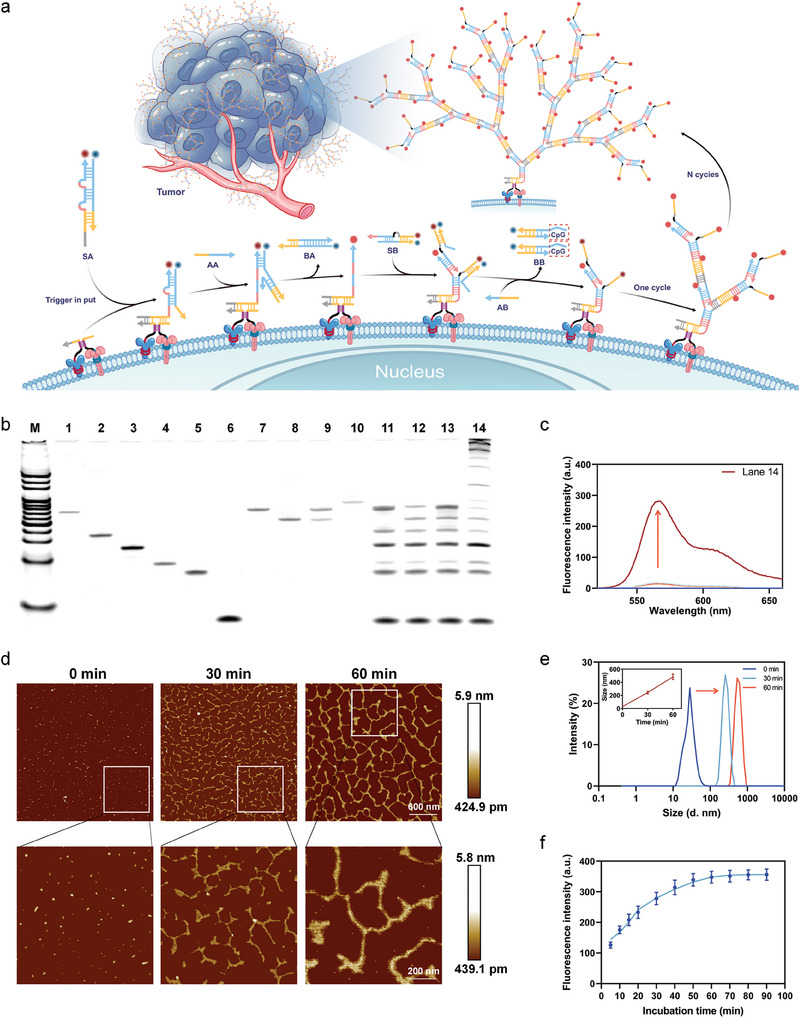
Characterization of the DNA nanotree. a) Scheme of fluorescent DNA nanotree self‐assembly process. b) Native PAGE analysis of the DNA nanotree self‐assembly. Lane M, 20 bp DNA ladder. c) Fluorescence analysis of Lanes 11–14. d) AFM images of DNA nanotree self‐assembly process for 0 min, 30 and 60 min. e) Diameter sizes of DNA nanotree self‐assembly process for 0, 30, and 60 min (n = 3), data are presented as mean ± SD. f) Fluorescence intensity of DNA nanotree self‐assembly process (n = 3), data are presented as mean ± SD.

The Panel‐HCR strategy for precise identification of tumor cells and regulation of TAMs reprogramming was divided into two modules: a recognition‐then‐assembly module and a release‐then‐regulation module. The recognition‐then‐assembly module was co‐triggered by combinatorial proteins on the membrane surface of tumor cells for DNA nano‐tree self‐assembly (**Figure** **1**a). Simultaneous recognition of the membrane surface proteins by the dual‐recognition probe led to significant increases in their hybridization melting temperature (Tm) and effective concentration, induced binding of the interaction region, and allowed the trigger sequences e and d to form an effective triggering single strand, which bound to the exposed toehold on SA to shift part of SAb from SAa and open the first loop of SA. The newly exposed SAb was replaced by AA from SA, and the second loop of SA was opened, releasing the by‐product BA. Thus, the quencher (BHQ2)‐labeled SAb separated from the fluorophore (Cy3)‐labeled SAa, accounting for fluorescence recovery. Subsequently, the exposed SAa with the same sequence could hybridize with the two SB toeholds at the same time. In the same manner as AA, AB displaced SBb from SB and produced the by‐product BB (the therapeutic chain for subsequent TAMs reprogramming). Similarly, two trigger chains with the same sequence as T were exposed to repeat the above chain substitution process numerous times, forming the highly branched, high molecular weight DNA nano‐tree. At the same time, more fluorescent groups were released to illuminate the cell surface. The release‐then‐regulation module was designed to inhibit tumor growth by regulating the transition of M2 TAMs into the M1 phenotype via BB, a CpG‐loaded byproduct released during DNA nano‐tree self‐assembly.

### 2.2 Feasibility of Recognition‐Then‐Assembly Module for Panel‐HCR

As a proof of concept, polyacrylamide gel electrophoresis (PAGE) and fluorescence spectroscopy were used to evaluate the feasibility of the as‐designed Panel‐HCR assembly. As shown in Figure S1a in the Supporting Information, the addition of the single‐chain trigger T revealed the appearance of a product with a lower mobility than SA (Lane 5), suggesting that the T chain could be docked at the SA‐exposed toehold. The addition of AA to the mixture accounted for the formation of SAb‐AA double‐stranded byproducts (BA) and T‐SAa double‐stranded (Lane 6). Lane 7 showed the appearance of products with even lower mobility, reflecting the binding of SB to T‐SAa double‐stranded. Finally, the introduction of AB triggered cyclic strand displacement to form highly branched, high molecular weight DNA nano‐trees (Lane 8). Therefore, it was shown that Panel‐HCR could significantly and progressively self‐assemble into high molecular weight DNA nano‐trees. It was also verified that almost no high molecular weight assembly products were observed in the absence of the trigger strand, indicating that the designed DNA nano‐tree self‐assembly system was highly specific and had excellent non‐reactive stability between remodeling probes (Figure S2a, Supporting Information). Fluorescence detection was carried out (Figure S1b and S2b, Supporting Information) to verify the DNA nano‐tree self‐assembly process and specificity.

To simulate the proximity hybridization at the cell membrane surface and the non‐linear HCR, a dummy chain (Ω chain) complementary to the aptamer sequence of the receptor‐recognition probe was synthesized (Figure 1b). In the absence of the Ω chain or receptor‐recognition probe (Lanes 11–13), a trigger chain could not be effectively formed to trigger the self‐assembly process. The formation of an initiator chain and the triggering of Panel‐HCR assemblage were only possible in the presence of both the Ω chain and the dual‐recognition probe (Lane 14). The above results were also confirmed by fluorescence spectroscopy (Figure 1c). In addition, atomic force microscopy (AFM) was used to directly image the self‐assembly products, offering further proof of PAGE results (Figure S3, Supporting Information). In the absence of the Ω chain, the dual‐recognition probe was unable to form the initiating chain efficiently, and only tiny spots could be observed on AFM images. In contrast, in the presence of the Ω chain, tree‐like structures of varying sizes appeared, suggesting that the DNA nano‐tree was generated by the dual‐targeted proteins being driven together. Subsequently, we controlled the reaction time to observe the DNA nano‐tree self‐assembly imaging, and it was seen that the 30‐min self‐assembly product was significantly smaller than the 60‐min self‐assembly product (Figure 1d), indicating the high efficiency and controllability of the present strategy. Dynamic light scattering (DLS, Figure 1e) measurements also confirmed these results. To further investigate the dynamics of DNA nano‐tree formation, the entire assembly process was explored by monitoring the fluorescence intensity (Figure 1f). As expected, strong fluorescence intensity was observed after 5 min, indicating the rapidity of the DNA nano‐tree self‐assembly process. As the reaction proceeded, the fluorescence intensity gradually increased and then stabilized after 60 min. This showed that after repeated chain displacement, the DNA nano‐tree gradually self‐assembled and grew in size.

Optimization of recognition‐then‐assembly module for Panel‐HCR. Proximity hybridization is a key step in the in situ imaging of the cell surface. The strategy employed in this study was to combine proteins on cell surfaces to bring two recognition probes close to each other, thereby significantly increasing their effective local concentration. This enabled the binding of interaction regions and the formation of trigger chains to effectively trigger DNA self‐assembly. To begin specific proximity hybridization, a series of oligonucleotides were synthesized to optimize the quantities of complementary base pairs in the interaction region of the recognition probe. Pairs of separated chains (Y1 and Y2) containing only the interaction regions (c and c*) and triggering regions (e and d), as well as proximity chains (HY12) connected to two separated chains Y through poly T sequences, were synthesized. When the interaction region was five bases long, the paired separated chains and the proximity chains did not bind to SA, and no proximity hybridization occurred (Figure S4, Supporting Information). However, when the interaction region was six or seven bases long, the proximity chains bound to SA, and the separated chains remained unbound. When the interaction region was eight or nine bases long, the paired separated chains spontaneously bound to SA. Thus, six and seven bases both fulfilled the requirement, but the use of seven bases was more stable, so an interaction region containing seven bases was used in the recognition probe. As shown in Figure 1b, EP (ace) and TP (bc *d) with a seven‐base interaction region did not exhibit non‐specific proximity hybridization in the separated state (Lane 9), indicating the successful triggering of non‐linear HCR using the selected proximity hybridization strategy.

### 2.3 In Vitro Imaging of The Recognition‐Then‐Assembly Module for Panel‐HCR

**Figure 2:**
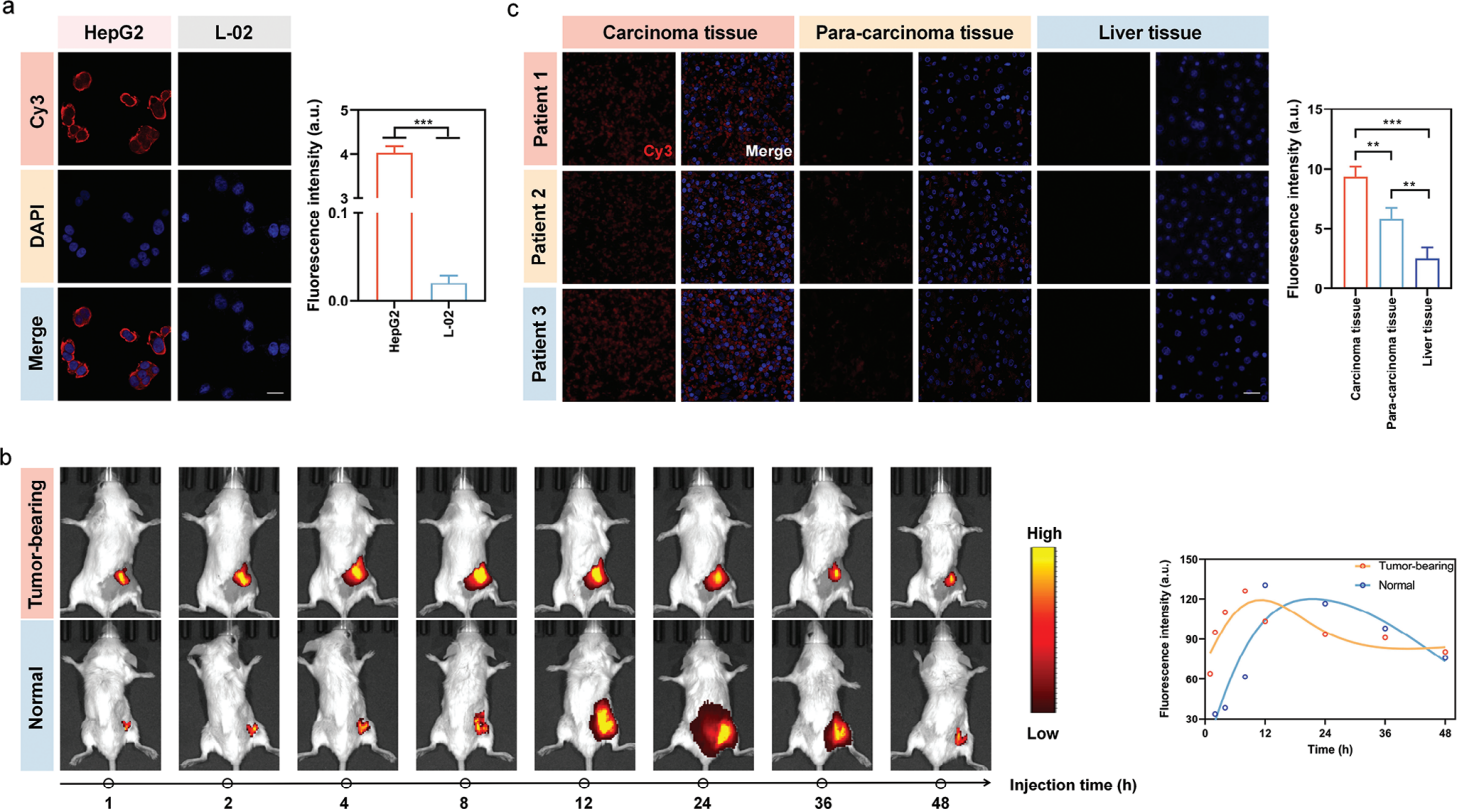
In vitro and in vivo imaging based on Panel‐HCR strategy. a) Imaging of HepG2 and L‐02 cells based on Panel‐HCR strategy, and the corresponding fluorescence intensities (n = 3), data are presented as mean ± SD, and significance is determined using Independent‐Samples T‐Tests. b) In vivo imaging study of tumor‐bearing and normal mice based on Panel‐HCR strategy, and the corresponding fluorescence intensity. c) In situ hybridization imaging of surgically resected carcinoma, paracancerous tissue and liver tissue from hepatocellular carcinoma patients based on Panel‐HCR strategy, and corresponding fluorescence intensity (n = 3), data are presented as mean ± SD, and significance is determined using ANOVA with Post Hoc Multiple Comparisons (LSD). Scale bar, 20 µm. **P < 0. 01, ***P < 0.001.

After ensuring the feasibility of the Panel‐HCR self‐assembly, the strategy was further studied in living cells. First, the specificity of the receptor‐recognition probe was verified. The receptor‐recognition probes EP (ace)‐Cy5.5 and TP (bc *d)‐FAM were individually or jointly incubated with hepatocellular carcinoma cells and L‐02 cells (negative control) and observed using a confocal laser scanning microscope (CLSM). Significant red and green fluorescence emissions were observed on hepatocellular carcinoma cells, whereas no noticeable fluorescence was seen in the control group. This means that there were almost no EpCAM molecules or TLS11a aptamer binding sites on the surfaces of the L‐02 cells, and this further demonstrates the specificity of the recognition probe in recognizing targets, as also verified by flow cytometry (Figures S5 and S6, Supporting Information). Amplification of the detected signal was required to better visualize the tumor cells. Panel‐HCR amplification was used to enhance the fluorescence signal. Incubating the Panel‐HCR strategy with tumor cells. As illustrated in **Figure** **2**a, the strong fluorescence signal was observed at the edges of the hepatocellular carcinoma cells but not the L‐02 cells. These results were cross‐verified with flow cytometry (Figure S7, Supporting Information). These observations confirm the applicability of the Panel‐HCR strategy to in situ imaging and visualization of tumor cell surfaces. In addition, we found that a single recognition receptor was unable to trigger DNA nano‐tree self‐assembly on the tumor cell surface, illustrating the ultra‐high specificity of the present strategy for triggering self‐assembly by combinatorial proteins (Figure S8, Supporting Information). To obtain the best imaging performance, the reaction time, receptor recognition probe, and substrate concentration of the DNA dendritic nano‐assembly were optimized; the optimal substrate SA concentration was chosen to be 200 nM, EP and TP concentrations were both 250 nM, and the reaction time was 60 min (Figure S9, Supporting Information). Next, we designed a control experiment without the aptamer region of the EP and/or TP (Figure S10, Supporting Information). The absence of either aptamer in EP and TP prevented red fluorescence from being seen on the cell membrane, indicating that EP or TP in the region of the missing aptamer could not recognize the corresponding specific site, suggesting that Panel‐HCR has good selectivity for the specific site of tumor cells.

### 2.4 In Vivo Imaging of The Recognition‐Then‐Assembly Module for Panel‐HCR

To test the image magnification ability of the strategy in tumor‐bearing mice, we constructed a subcutaneous hepatocellular carcinoma model in BALB/c mice. The recognition probes and Cy5.5‐labeled remodeling probes were injected into the tumor sites of tumor‐bearing mice, and the time‐dependent, whole‐body fluorescence was measured. In tumor‐bearing mice, the fluorescence intensity gradually increased, peaking at 8 h. In normal mice (control), the fluorescence was weaker in the first 8 h. This suggests that the DNA nano‐tree self‐assembly occurred in the first 8 h in the tumor‐bearing mice, whereas almost no DNA nano‐trees were produced in the normal mice, suggesting that specific DNA nano‐tree self‐assembly could only occur in the presence of the target tumor cells (Figure 2b). The above results pointed to the significant potential of the proposed strategy in the specific recognition of tumor cells and in situ imaging in living animals. In situ hybridization and imaging were performed on the pathological tissues. Panel‐HCR was hybridized in situ with the liver tissue, paracancerous tissue, and cancer tissue of three hepatocellular carcinoma patients who had undergone surgery (Figure 2c). Strong red fluorescence from Cy3 was seen in the cancer tissue. Weak Cy3 fluorescence was seen in the paracancerous tissue, and an even weaker Cy3 fluorescence was observed in the normal liver tissue. The fluorescence intensity in the cancer tissue and paracancerous tissue was 3.67‐fold (*P* < 0.001) and 2.29‐fold (*P* < 0.001) than that of the normal liver tissue, respectively. Most importantly, our Panel‐HCR strategy achieved in situ imaging of tumor cells through in situ hybridization of pathological tissues and enabled tumor and non‐tumor tissues to be distinguished.

### 2.5 Release‐Then‐Regulation Module of Panel‐HCR for Reprogramming TAMs

**Figure 3:**
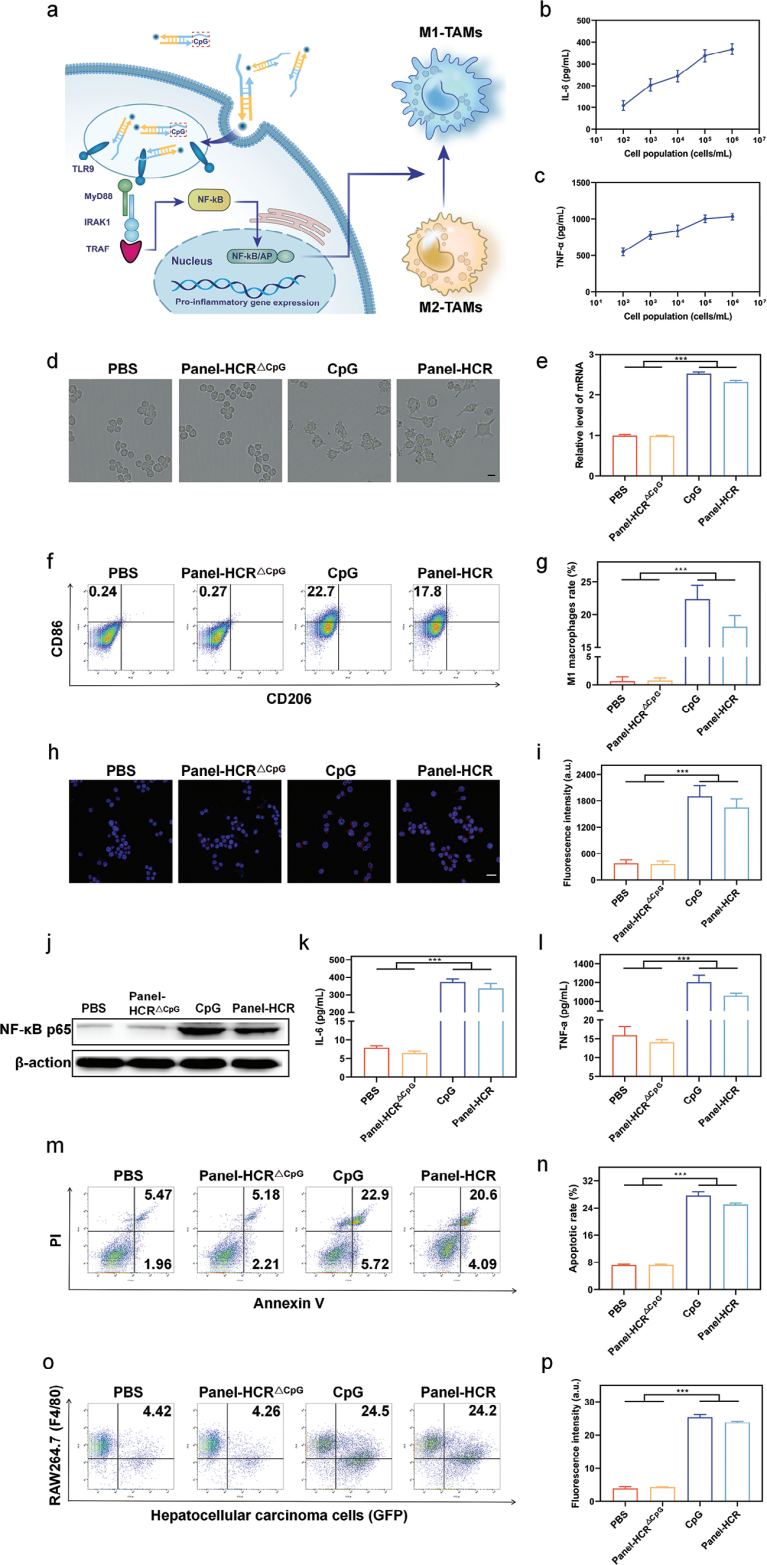
Panel‐HCR strategy has a strong ability to induce polarization of TAMs towards M1 phenotype. a) Mechanism diagram of Panel‐HCR strategy inducing macrophage polarization through activation of the NF‐κB signaling pathway. b,c) Relationship between pro‐inflammatory cytokine release (IL‐6, TNF‐α) and tumor cell number (n = 3), data are presented as mean ± SD. d) Morphology of RAW264.7 cells treated with Panel‐HCR strategy. Scale bar, 40 µm. e) The TLR9 expression of RAW264.7 cells treated with Panel‐HCR strategy (n = 3), data are presented as mean ± SD, and significance is determined using ANOVA with Post Hoc Multiple Comparisons (LSD). f,g) The CD86 (a marker for M1 TAMs) expression treated with Panel‐HCR strategy, and corresponding percentages (n = 3), data are presented as mean ± SD, and significance is determined using ANOVA with Post Hoc Multiple Comparisons (LSD). h,i) The iNOS expression (red fluorescence) of RAW264.7 cells treated with Panel‐HCR strategy. Blue fluorescence, nucleus (n = 3), data are presented as mean ± SD, and significance is determined using ANOVA with Post Hoc Multiple Comparisons (LSD). Scale bar, 20 µm. j) The western blot images of NF‐κB and p65 expression of RAW264.7 cells treated with Panel‐HCR strategy. k,l) Secretion of pro‐inflammatory cytokines IL‐6 and TNF‐α of RAW264.7 cells treated with Panel‐HCR strategy (n = 3), data are presented as mean ± SD, and significance is determined using ANOVA with Post Hoc Multiple Comparisons (LSD). m,n) Apoptosis analysis of hepatoma carcinoma cell and corresponding apoptosis percentage (n = 3), data are presented as mean ± SD, and significance is determined using ANOVA with Post Hoc Multiple Comparisons (LSD). o, p) Macrophage phagocytosis assessment (n = 3), data are presented as mean ± SD, and significance is determined using ANOVA. ***P < 0.001.

The above experiments demonstrated the feasibility and high specificity of the proposed strategy in identifying tumor cells and enabling in situ visual imaging of cell membrane surfaces. During the self‐assembly of the DNA nano‐tree, a byproduct BB strand was constantly shed, and the free end of the shed BB strand was the CpG sequence undergoing thiolation modification. Before performing the experiments, the relationship between the amount of CpG released and the number of tumors was explored. Hepatocellular carcinoma cells (upper chamber) and RAW264.7 cells (lower chamber) were co‐cultured in a transwell system, and the Panel‐HCR strategy was performed for 24 h. Cytokine secretion of the supernatant was detected. As shown in **Figure** **3**b,c, interleukin 6 (IL‐6) and tumor necrosis factor α (TNF‐α) were positively correlated numbers of tumor cells, indicating that the amount of CpG release was dependent on the number of tumor cells. Next, the effect of the Panel‐HCR strategy on the polarization of RAW264.7 cells was investigated. The cells were divided into four groups: PBS (negative control), Panel‐HCR^△CpG^ (nano‐tree self‐assembly without CpG release), CpG (positive control), and Panel‐HCR groups.

First, a CCK8 assay was used to evaluate the cytotoxicity of each treatment on hepatocellular carcinoma cells and RAW264.7 cells (Figure S11, Supporting Information), which was found to be negligible. Next, the SBb chain (loaded with CpG) was labeled with FAM fluorescent groups (emitting green fluorescence), as shown in Figure S12 in the Supporting Information. RAW264.7 cells and hepatocellular carcinoma cells were co‐cultured, and the Panel‐HCR strategy was adopted for processing. As the reaction proceeded, an increasing number of SBb chains with fluorescent groups were observed in the RAW264.7 cells, with the strongest fluorescence measured at 24 h. This indicated enrichment of the thio‐modified CpG chains in RAW264.7 cells.

It is well recognized that RAW264.7 cells in their original state show no activity and adopt the M0 phenotype with a rounded shape (Figure 3d). After co‐cultivation, however, the CpG‐treated group and the Panel‐HCR‐treated group each had a strong inductive effect on the morphological changes in these cells, stimulating cell body enlargement and the growth of pseudopodia. Such changes were not observed in the PBS‐treated group or the Panel‐HCR^△CpG^‐treated group. The strong polarization‐inducing effect of Panel‐HCR leading to the transformation of RAW264.7 into M1 TAMs was quantitatively verified. The expression of M1 TAM marker CD86 increased by 17.47% (Figure 3f,g), and the expression of CD80 increased 2.24‐fold (Figure S13, Supporting Information). M1 TAM‐specific marker‐inducible nitric oxide synthase (iNOS) was elevated 4.33‐fold in Panel‐HCR‐treated RAW264.7 cells (Figure 3h,i). In addition, ELISA kits were used to check for IL‐6 and TNF‐α in the supernatant. By comparison with the PBS‐treated group, the Panel‐HCR‐treated group secreted significantly higher amounts of TNF‐α (66.74 times higher) and IL‐6 (42.56 times higher) (Figure 3k,l), indicating the excellent polarization‐inducing power of Panel‐HCR.

To demonstrate the anti‐tumor capability of Panel‐HCR, RAW264.7 cells (upper chamber) and hepatocellular carcinoma cells (lower chamber) were co‐cultured for 24 h, and cell apoptosis was measured using flow cytometry. The Panel‐HCR group showed a significantly higher cell apoptosis rate (17.90% higher) compared with the PBS group (Figure 3m,n). The result attested to the ability of Panel‐HCR to effectively promote tumor cell apoptosis and serve as an efficient anti‐tumor strategy. Subsequently, treated RAW264.7 cells and green fluorescent protein (GFP) transfected hepatocellular carcinoma cells were co‐incubated to verify the phagocytosis capacity of M1 TAMs in vitro. It was demonstrated that the phagocytic index of RAW264.7 cells phagocytizing Hepa1‐6 cells in Panel‐HCR‐treated group was elevated by 19.86% in comparison to that in the PBS‐treated group (Figure 3o,p).

Next, the action mechanism of the Panel‐HCR strategy was investigated. CpG was found to trigger the release of pro‐inflammatory cytokines by binding to TLR9 receptors and activating the NF‐κB signaling pathway (Figure 3a). A 2.32‐fold increase in TLR9 levels was observed in the Panel‐HCR‐treated group (Figure 3e), and the level of the key downstream molecule p65 of the NF‐κB signaling pathway also increased significantly (Figure 3j). These results suggest that the Panel‐HCR strategy utilizes the NF‐κB signaling pathway to induce polarization in RAW264.7 cells.

**Figure 4:**
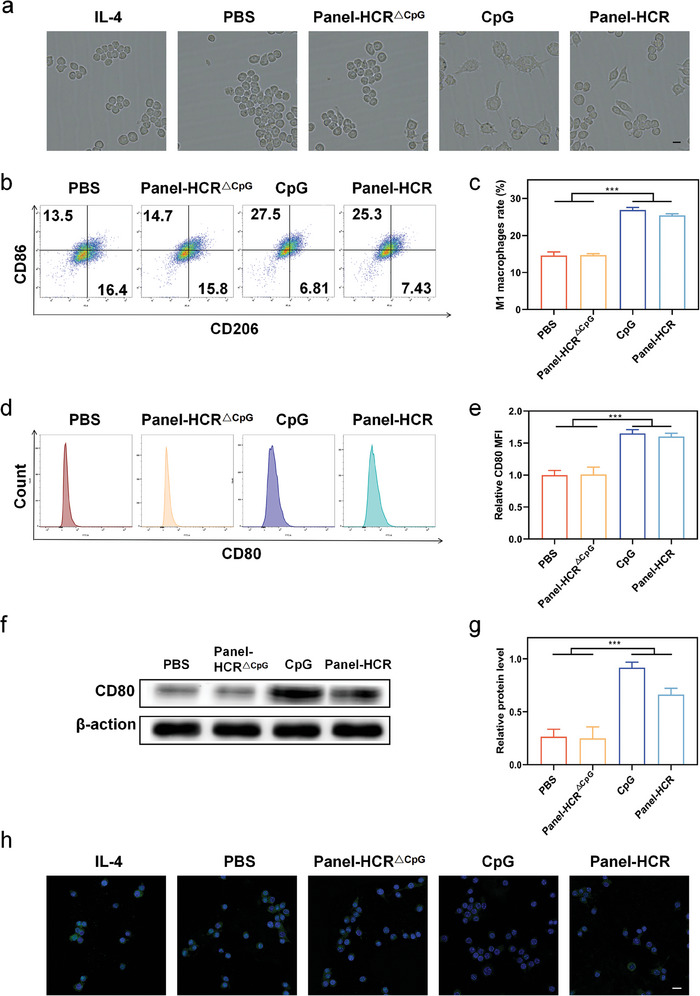
Panel‐HCR strategy induces repolarization of M2 TAMs toward the M1 phenotype. a) Morphology of M2 TAMs repolarization toward the M1 phenotype treated with Panel‐HCR strategy. Scale bar, 40 µm. b,c) The CD86 (M1 TAMs marker) and CD206 (M2 TAMs marker) expression pretreated with IL‐4 and then treated with Panel‐HCR strategy, and corresponding percentages (n = 3), data are presented as mean ± SD, and significance is determined using ANOVA with Post Hoc Multiple Comparisons (LSD). d,e) The CD80 (M1 TAMs marker) expression pretreated with IL‐4 and then treated with Panel‐HCR strategy, and corresponding percentages (n = 3), data are presented as mean ± SD, and significance is determined using ANOVA with Post Hoc Multiple Comparisons (LSD). f,g) Western blot of CD80 (M1 TAMs marker) expression, and the corresponding percentages (n = 3), data are presented as mean ± SD, and significance is determined using ANOVA with Post Hoc Multiple Comparisons (LSD). h) The Arg‐1 (green fluorescence) expression of RAW264.7 cells pretreated with IL‐4 and then treated with Panel‐HCR strategy. Blue fluorescence, nucleus. Scale bar, 20 µm. ***P < 0.001.

Immunosuppressive M2 TAMs are generally present in hepatocellular carcinoma. The inductive ability of Panel‐HCR in the polarization of M2 TAMs to M1 TAMs was investigated. First, RAW264.7 cells were treated with 20 ng/mL of interleukin 4 (IL‐4). The treated cells were then co‐cultured with hepatocellular carcinoma cells. The RAW264.7 cells pretreated with IL‐4 (to induce M2 TAM formation) appeared oval in shape, whereas the CpG group and Panel‐HCR group showed irregular shapes and the growth of pseudopodia (**Figure** **4**a). The PBS‐treated group and Panel‐HCR^△CpG^‐treated group did not show any significant changes. These results attest to the good polarization‐inducing ability of the Panel‐HCR strategy. This effect of Panel‐HCR was quantitatively studied next. The number of CD86^+^ CD206^−^ cells (M1 TAMs) in the Panel‐HCR‐treated group increased from 14.57% to 25.43%, whereas the number of CD86^−^ CD206^+^ cells (M2 TAMs) decreased from 15.67% to 7.19% (Figure 4b,c; Figure S14, Supporting Information). The expression of the M1 TAM marker CD80 was 1.60 times higher in the Panel‐HCR‐treated group compared with PBS‐treated group (Figure 4d–g), and the expression of the M2 TAM‐specific marker arginase 1 (Arg‐1) in the Panel‐HCR‐treated group accounted for 39.5% of the PBS‐treated group (Figure 4h; Figure S15, Supporting Information). These results demonstrate the strong ability of Panel‐HCR to promote the polarization of M2 TAMs to M1 TAMs.

**Figure 5:**
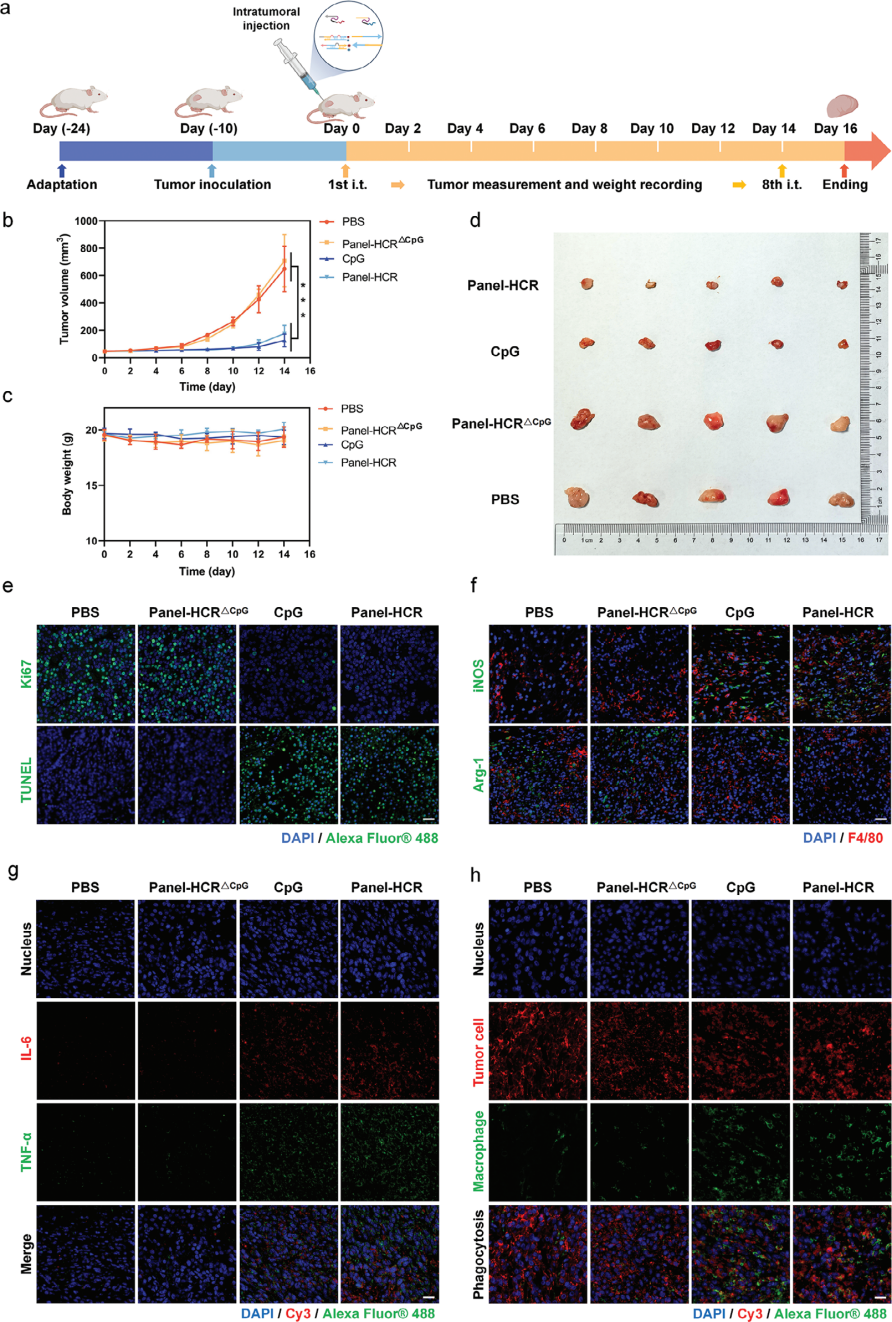
Immunotherapy of Panel‐HCR strategy for tumor‐bearing mice. a) Schematic diagram of immunotherapy for tumor‐bearing mice. b,c) Recording of tumor volume and body weight of tumor‐bearing mice during treatment (n = 5). d) Photographs of tumors resected of each group at the end of treatment. e) Ki67 immunofluorescence (upper panel) and TUNEL staining (lower panel) of tumor tissue sections at the end of treatment. Scale bar, 20 µm. f) Immunofluorescence double staining analysis of iNOS (upper panel) and Arg‐1 (lower panel) expression in tumor tissue sections at the end of treatment. Scale bar, 20 µm. ***P < 0.001. g) Immunofluorescence double staining analysis of TNF‐α and IL‐6 levels in tumor tissue sections at the end of treatment. Scale bar, 20 µm. h) Immunofluorescence double staining analysis of macrophage phagocytosis in tumor tissue sections at the end of treatment. Scale bar, 20 µm.

In vivo immunotherapeutic effect of release‐then‐regulation module of Panel‐HCR. On the basis of the above in vitro anti‐tumor studies, the immunotherapeutic effect of Panel‐HCR in the body was investigated. BALB/c mice were treated following the timetable shown in **Figure** **5**a. The 10 days after subcutaneous tumor implantation, the mice were randomly divided into four groups, which received either PBS (negative control), Panel‐HCR^△CpG^ (nano‐tree self‐assembly without CpG release), CpG (positive control), or Panel‐HCR via intratumoral injection. The injection was performed every other day for eight injections in total. Mouse weight and tumor volume were measured every other day after injection. Rapid tumor growth was identified in the PBS group and Panel‐HCR^△CpG^ group, whereas significantly inhibited tumor growth was observed in the other two groups (Figure 5b). This indicated the strong abilities of Panel‐HCR to remodel the TME, promote TAMs polarization, and significantly inhibit the growth of tumor cells. It was also noticed that there was no significant change in body weight across the groups during the treatment period (Figure 5c). After 16 days of treatment, the tumor volume in each tumor‐bearing group was measured and was found to be no more than 1000 mm^3^. The biosafety of the treatment procedures was assessed by quantitatively measuring the hematological and biochemical indices of each group. No observable abnormalities in blood test results or renal function were detected, but abnormally high values were noticed in liver function indicators, which could be due to the hepatocellular carcinoma tissue (Figure S16, Supporting Information). Interestingly, the Panel‐HCR group and CpG group showed improved liver dysfunction. Hematoxylin and eosin (H&E) staining was performed on key organs (Figure S17, Supporting Information). No noticeable lesions were found in the spleen, heart, liver, kidneys, or lungs in any of groups, and the tissues retained their structural integrity. After treatment, the mice were euthanized, and the tumor was removed to measure its volume (Figure 5d). It was concluded that the Panel‐HCR strategy could effectively inhibit tumor growth.

To further evaluate the effectiveness of this immunotherapy, immunofluorescence assay was performed on the removed tumor tissues in each group (Figure 5e). Consistent with tumor growth, Ki67 staining showed a significant reduction in positive tumor cells in both Panel‐HCR‐ and CpG‐treated groups. Terminal dUTP nick‐end labeling (TUNEL) staining showed a significant increase in positive cells in both Panel‐HCR‐ and CpG‐treated groups. These results fully demonstrate which the Panel‐HCR strategy has the best ability to inhibit tumor growth and promote tumor cell apoptosis in vivo. Double immunofluorescence labeling was performed next. Compared with the negative control group, a significant increase in the M1 marker (iNOS) was observed in the Panel‐HCR‐treated group, and a decrease was observed in the M2 marker (Arg‐1) (Figure 5f). These results suggest that the Panel‐HCR strategy is effective in converting infiltrating M2 TAMs into the M1 phenotype in TME, providing evidence for its applicability to in vivo immunotherapy. To further verify the mechanism of tumor suppression in vivo, immunofluorescence labeling was performed on tumor tissue sections after treatment. More pro‐inflammatory factors TNF‐α and IL‐6 in the Panel‐HCR and CpG‐treated group have secreted to the TME, in contrast to those in PBS and Panel‐HCR^△CpG^ group which was consistent with the in vitro experiments (Figure 5g). At the same time, a large number of proliferative CD4^+^ T cells and CD8^+^ T cells were clearly seen (Figure S18, Supporting Information). These results indicated that the Panel‐HCR strategy could promote the secretion of inflammatory factors and activate T cells to exert a greater killing effect on tumors. In addition, the phagocytic ability of M1 TAMs phagocyting tumor cells was also probed (Figure 5h). The numbers of M1 TAMs of tumors in the Panel‐HCR and CpG‐treated groups were much higher than those in the PBS and Panel‐HCR^△CpG^‐treated groups.

## 3 Discussion

To equilibrate the balance between the populations of tumor cells and immunoactivity of polarized TAMs in TME, we established a smart tumor cell‐derived Panel‐HCR strategy for on‐demand TAM reprogramming. Our design was composed of two inseparable modules: a recognition‐then‐assembly module and a release‐then‐regulation module. In module one, the bispecific recognition of tumor cell‐specific proteins on cell surfaces could trigger Panel‐HCR, leading to the assembly of a DNA nano‐tree to achieve cell‐surface bioimaging of single cells, tissues, and living mice. Meanwhile, the preloaded CpG ODNs, as byproduct strands of the DNA nano‐tree assembly process, were released to the TME. Notably, the release of CpG‐ODNs relies on the populations of recognized tumor cells in a concentration‐dependent manner, meaning the release is truly on‐demand. Subsequently, the released CpG‐ODNs, serving as the link between the two modules, efficiently regulate the immunoactivity of TAMs and repolarize M2 TAMs into the M1 phenotype in module two. This on‐demand regulation could induce the release of pro‐inflammatory cytokines by binding to TLR9 receptors and activating the NF‐κB signaling pathway. In an example of positive feedback, the repolarized M1 TAMs exerted cytotoxic activity on targeted tumor cells in vitro and in vivo without notable side effects. To the best of our knowledge, this is the first successful attempt to modulate the population‐immunoactivity relationship of tumor cells and TAMs in TME to ultimately realize antitumor immunotherapy via DNA nanotechnology. We believe that this proposed strategy could be extended to recognize other cell‐surface biomarkers and release other immunomodulators, thus providing a new paradigm for modulating immunosuppressive TME and developing antitumor therapeutics.

## Conflict of Interest

The authors declare no conflicts of interest.

## Supporting information

Supporting Information

## Data Availability

The data that support the findings of this study are openly available in Kai Chang at DOI, reference number 50.
