# Supplementary material for: Smart Tumor Cell‐Derived DNA Nano‐Tree Assembly for On‐Demand Macrophages Reprogramming
Source: Adv Sci (Weinh). 2023 Dec 25;11(10):2307188. doi: 10.1002/advs.202307188 (PMC10933644; doi:10.1002/advs.202307188)
Supplement: Supplementary file 1 — Supporting Information [file ADVS-11-2307188-s001.pdf]

ADVANCED SCIENCE
Open Access

Supporting Information

for *Adv. Sci.*, DOI 10.1002/advs.202307188

Smart Tumor Cell-Derived DNA Nano-Tree Assembly for On-Demand Macrophages Reprogramming

Zhiguo Chen, Sha Yang, Zhuyang Zhao, Liu Feng, Jing Sheng, Ruijia Deng, Binpan Wang, Yuan He, Dan Luo, Ming Chen, Lei Chen* and Kai Chang**

Supplementary information

Smart Tumor Cell-Derived DNA Nano-tree Assembly for On-Demand Macrophages Reprogramming

Zhiguo Chen[1,2#], Sha Yang[1#], Zhuyang Zhao[1], Liu Feng[1], Jing Sheng[1], Ruijia Deng[1], Binpan Wang[1], Yuan He[1], Dan Luo[3], Ming Chen[1], Lei Chen[2*], Kai Chang[1*]*

1. Department of Clinical Laboratory Medicine, Southwest Hospital, Army Medical University (Third Military Medical University), 30 Gaotanyan, Shapingba District, Chongqing 400038, China

2. Department of Gastroenterology, Southwest Hospital, Army Medical University (Third Military Medical University), 30 Gaotanyan, Shapingba District, Chongqing 400038, China

3. Department of Biological and Environmental Engineering, Cornell University, Ithaca NY14853-5701, USA

Corresponding Author

* Kai Chang

Email: changkai0203@163.com

* Lei Chen

Email: chenlei1977603@126.com

* Ming Chen

Email: chming1971@126.com

[#] Zhiguo Chen and Sha Yang contributed equally to this work.

Experimental sections:

Materials. All DNA oligonucleotides (Table S1, Supporting Information) were synthesized and obtained from Sangon Biotech Co., Ltd. (China). FBS, RPMI-1640 medium, and DMEM were obtained from VivaCell (China). CCK-8 cell proliferation kit and 5% BSA were purchased from Solarbio (China). Hoechst33342 and anti-mouse CD206-PE antibody were obtained from Invitrogen (USA). The Annexin V-FITC kit was bought from Sigma-Aldrich Co. (USA). The penicillin/streptomycin solution was bought from Gibco (USA). The horseradish peroxidase (HRP)-conjugated anti-rabbit IgG secondary antibodies were obtained from Biospes Co., Ltd. (China). The anti-NF-κB p65 antibody, anti-Arg-1 antibody, and anti-iNOS antibody were bought from Abcam (UK). Mouse TNF-α Valukine ELISA Kit, Mouse IL-6 Valukine ELISA kits, and Anti-mouse CD86-AF488 antibody were obtained from NOVUS (USA). The anti-mouse CD80-FITC antibody was bought from BioLegend (USA). The female BALB/c mice (n = 20, 4-6 weeks old) were purchased from Hunan SJA Laboratory Animal Co. We conducted all animal experiments in strict accordance with the Guidelines for the Care and Use of Laboratory Animals (Approval No. AMUWEC20223583), which was approved by the Laboratory Animal Welfare and Ethics Committee of the Third Military Medical University. Liver tissues, paracancerous tissues, and cancerous tissues surgically resected from three patients with hepatocellular carcinoma obtained from the hospital were approved for use by the Ethics Committee of the Southwest Hospital of the Third Military Medical University (Approval No. KY2020146). Informed written consent was also provided by all participants.

Design of recognition-then-assembly module for Panel-HCR. Cy3-labeled probe SAa was mixed with BHQ2-labeled probe SAb (1:1.5 molar ratio) at 95°C for 5 min

and gradually cooled to room temperature to form intact probe SA. A similar method was utilized to obtain probe SB. The Panel-HCR was formed by self-assembly of SA, SB, AA, AB, Ω, EP, and TP with a 1:2:2:4:0.5:0.5:0.5:0.5:0.5 molar ratio. The formation of Panel-HCR was verified using 12% PAGE. Size was measured using Zetasizer. The formed Panel-HCR strategy was then scanned by fluidic mode AFM (NanoWizard BioAFM, USA) on pre-treated dry mica substrates.

Atomic force microscopy (AFM). Reaction products were diluted the same number of times, placed on freshly cleaved mica for 5 min, and then dried with nitrogen. Images were acquired using a NanoWizard BioAFM (Bruker, USA), and the image background was processed with Nanoscope 2.0 software.

Fluorescence in situ hybridization. Tissues were fixed in 4% paraformaldehyde, sequentially subjected to gradient dehydration, paraffin embedding, tissue sectioning, and proteinase K (20 μg/mL) digestion for 15 min, incubated with the Panel-HCR strategy at 42°C overnight, and observed by fluorescence microscopy.

Cellular cytotoxicity assays. RAW264.7 cells and hepatocellular carcinoma cells were inoculated in 96-well plates with 5×10^3 cells and 100 mL of medium per well. After treated with the Panel-HCR strategy for different times (2, 4, 8, 12, 24, 48, and 72 h), the medium was replaced with fresh medium, and 10 μL of CCK-8 was added to the cells and incubated for 4 h. The cells were incubated for 4 h using the Thermo Scientific Varioskan Flash (USA) to detect the absorbance at 450 nm.

Cellular uptake. Co-culture was performed in the above manner, and after Panel-HCR strategy treatment for different times (1, 6, 12, and 24 h), RAW264.7 cells were collected and inoculated on confocal dish for 4 h, protected from light, fixed (with 4% tissue fixative), and then the nuclei were treated with DAPI for 15 min. Finally, the images were captured using a laser confocal microscope (Zeiss LSM780,

Germany). Only SBb was labeled with the FITC fluorescent moiety.

Cell apoptosis assay. Hepatocellular carcinoma cells collected with the above-methods were incubated with 1 μL of propidium iodide and 5 μL of annexin-V in the dark for 15 min at room temperature, and apoptosis of hepatocellular carcinoma cells was detected by using a BD FACSCanto II flow cytometer (USA).

Confocal laser scanning microscopy imaging. RAW264.7 cells treated with the Panel-HCR strategy were inoculated into confocal dishes for 4 h, protected from light, fixed (with 4% tissue fixative), broken, sealed, treated with DAPI for 15 min, incubated overnight with the primary antibody, incubated with the secondary antibody for 50 min at room temperature, and finally imaged.

Macrophage phagocytosis assessment. Hepatocellular carcinoma cells were stabilized to express green fluorescent protein by lentiviral infection. RAW264.7 cells (lower chamber) and hepatocellular carcinoma cells (upper chamber) were co-cultured in a transwell system. After four treatments for 24 h, RAW264.7 cells were collected on a six-well plate, and hepatocellular carcinoma cells were added to the treated RAW264.7 cells (1:1). With co-incubation for 5 h, the unfixed hepatocellular carcinoma cells were washed using PBS, the adherent cells in the six-well plate were collected, centrifuged and resuspended. At last, the RAW264.7 cells were labeled using PE-F4/80 for 40 min and detected using flow cytometry.

In vitro macrophage polarization marker assay. Hepatocellular carcinoma cells (upper chamber) and RAW264.7 cells (lower chamber) were co-cultured in a Transwell system (USA). Then, the Panel-HCR strategy was added to the upper chamber. After 24 h of incubation, the cells in the lower chamber were collected, resuspended in 400 mL of PBS, blocked in 2% BSA for 30 min, washed with cold

PBS, and incubated with anti-CD206 and anti-CD86 for 30 min in the dark. Finally, the flow cytometry was performed on the cells.

Establishment of tumor-bearing mice model and animal experiment. Mice were anesthetized using isoflurane, and hepatocellular carcinoma cells (10^6 cells) were injected subcutaneously into the right buttock of each mouse. Tumor growth was measured using vernier calipers, and the tumor volume was calculated as $l \times w^2/2$ (where l is the long axis and w is the short axis). Ten days after subcutaneous injection of hepatocellular carcinoma cells, the mice were randomly divided into four groups: PBS (negative control), Panel-HCR$^{\triangle CpG}$ (nanotree self-assembly without CpG release), CpG (positive control), and Panel-HCR groups, and intratumor injections were administered every two days for a total of eight injections. The body weight and tumor volume of the mice were recorded throughout the treatment. The *in vitro* imaging fluorescence of mice was monitored and analyzed with IVIS Spectrum CT over time (1, 2, 4, 8, 12, 24, 36, and 48 h) to understand the imaging changes of subcutaneous tumors by the Panel-HCR strategy. At the end of the eight treatments, the mice were anesthetized and killed. Blood was taken from the eyes for biochemical and hematological examinations. Tumors were removed at the end of the treatment for photographic documentation. Tumor tissue sections were subjected to Ki67 immunofluorescence and TUNEL staining to assess proliferation and apoptosis of tumor cells. Finally, routine histologic examinations of the heart, liver, spleen, lungs, and kidneys of the mice were performed.

Statistical analysis. All data in this study are expressed as mean ± standard deviation (SD), based on three independent experiments. Statistical differences between two groups were compared using Independent Sample T-tests, and comparisons between multiple groups were performed using one-way ANOVA. P <

0.05 was considered statistically significant.

Table S1. DNA sequences and modifications.

DNA Name	Sequence (5'- 3')
Sequences of Panel-HCR	
SAa	Cy3-GTGCTCCATGACGTTCCT**GACGTT**CTCCTGTGCTCC ATGACGTTCCT**GACGTT**CTCCTCAGCTTCATCAACTAGT TCGTCA
SAb	AACTAGTTGATGAAGCTGAGGAACGTCATGGAGCACAG GAACGTCATGGAGCAC-BHQ2
AA	CTCCATGACGTTCCTGTGCTCCATGACGTTCCTCAGCTT
SBa	AGGAGA*A*C*G*T*C*A*G*G*A*A*C*G*T*C*A*T*G*G* AGCACAAAATGACGAACTAGTTGATGAAGCTG-Cy3
SBb	BHQ2-CAGCTTCATCAACTAGTTGTGCT*C*C*A*T*G*A*C *G*T*T*C*C*T*G*A*C*G*T*T
AB	GCACAACTAGTTGATGAAGC
Ω	CAGCACGTCCATGAGGCGGAAACCGTAACAATCACAAT GCGATTGTTCACATGGGGATGCTGTTTTTTTTTTTTTTT TTTTTTTTTTTTTTTCAGGCCAACCCCCCATGACAACGT GGGACAGACGCAACCTCTGTAGTG

EP	CACTACAGAGGTTGCGTCTGTCCCACGTTGTCATGGGGGGTTGGCCTGTTTTTTTTTTTTTTTTTTTTTTTTTTTTTTTTTTTTTTTTTTCACTCCTTTTGATGAAGCTG
TP	TGACGAACTAGTTGGAGTGATTTTTTTTTTTTTTTTTTTTTTTTTTTTTTTTTTTTTTTTTACAGCATCCCCATGTGAACAATCGCATTGTGATTGTTACGGTTTCCGCCTCATGGACGTGCTG
T	TGACGAACTAGTTGATGAAGCTG

Sequences of Panel-HCR$^{\triangle CpG}$

SAa	GTGTGCCTATTATGTC**TC**CTCCTGTGTGCCTATTATGTC**TC**CTCCTCAGCTTCATCAACTAGTTCGTCA
SAb	AACTAGTTGATGAAGCTGGACATAATAGGCACACGACATAATAGGCACAC
AA	GTGCCTATTATGTCGTGTGCCTATTATGTCCAGCTT
SBa	AGGAGGAGACATAATAGGCACACTGACG**AA**CTAGTTGATGAAGCTG
SBb	CAGCTTCATCAACTAGGTGTGCCTATTATGTCTC
AB	GCACACCTAGTTGATGAAGC
Ω	CAGCACGTCCATGAGGCGGAAACCGTAACAATCACAATGCGATTGTTCACATGGGGATGCTGTTTTTTTTTTTTTTTTTTTTTTTTTTTTTTTTCAGGCCAACCCCCCATGACAACGTGGGACAGACGCAACCTCTGTAGTG

EP	CACTACAGAGGTTGCGTCTGTCCCACGTTGTCATGGGGG<br>GTTGGCCTGTTTTTTTTTTTTTTTTTTTTTTTTTTTTTTTTT<br>TTTTTTTTCACTCCTTTTGATGAAGCTG
TP	TGACGAACTAGTTGGAGTGATTTTTTTTTTTTTTTTTTTTTT<br>TTTTTTTTTTTTTTTTTTTTACAGCATCCCCATGTGAACAAT<br>CGCATTGTGATTGTTACGGTTTCCGCCTCATGGACGTGCT<br>G
Primer sequences for quantitative real-time PCR.	
TLR9	Forward primer: GGACGGGAACTGCTACTACAAGA<br>Reverse primer: TGGTCACAGCGACGGCAAT
CpG	T*C*C*A*T*G*A*C*G*T*T*C*C*T*G*A*C*G*T*T

* denotes thiosulfate modification

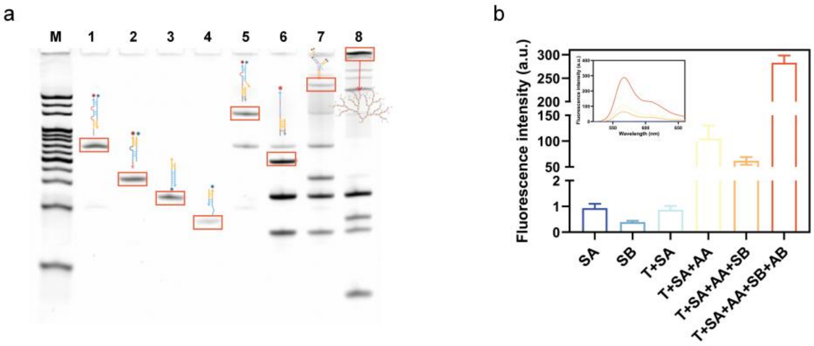

Figure S1. Native PAGE validation of DNA nanotree self-assembly and fluorescence analysis (Lanes 1-8: SA, SB, BA, BB, T+SA, T+SA+AA, T+SA+AA+SB, T+SA+AA+SB+AB) (n=3), data are presented as mean ± SD.

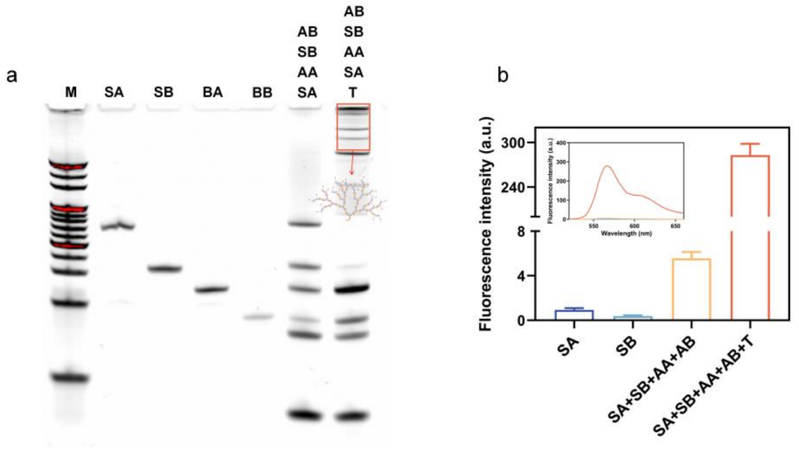

Figure S2. Native PAGE validation of the specificity of DNA nanotree self-assembly and fluorescence analysis (Lanes 1-6: SA, SB, BA, BB, SA+AA+SB+AB, T+SA+AA+SB+AB) (n=3), data are presented as mean ± SD.

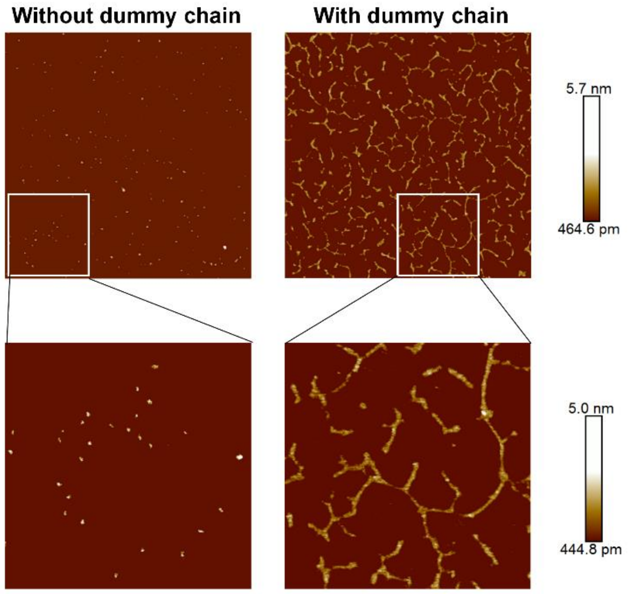

Figure S3. AFM imaging of Panel-HCR without and with the dummy chain.

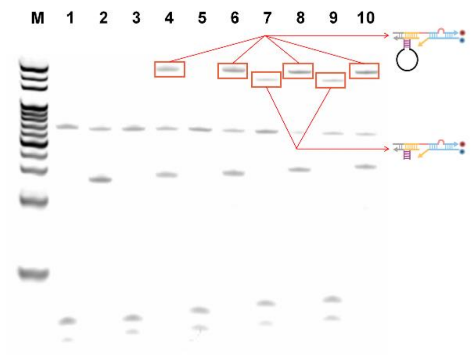

Figure S4. Panel-HCR optimization: PAGE analysis of the reaction products of SA with Y1-5 and Y2-5 (Lane 1), HY12-5 (Lane 2), Y1-6 and Y2-6 (Lane 3), HY12-6 (Lane 4), Y1-7 and Y2-7 (Lane 5), HY12-7 (Lane 6), Y1-8 and Y2-8 (Lane 7), HY12-8 (Lane 8), Y1-9 and Y2-9 (Lane 9), HY12-9 (Lane 10) after incubation for 60 min, respectively.

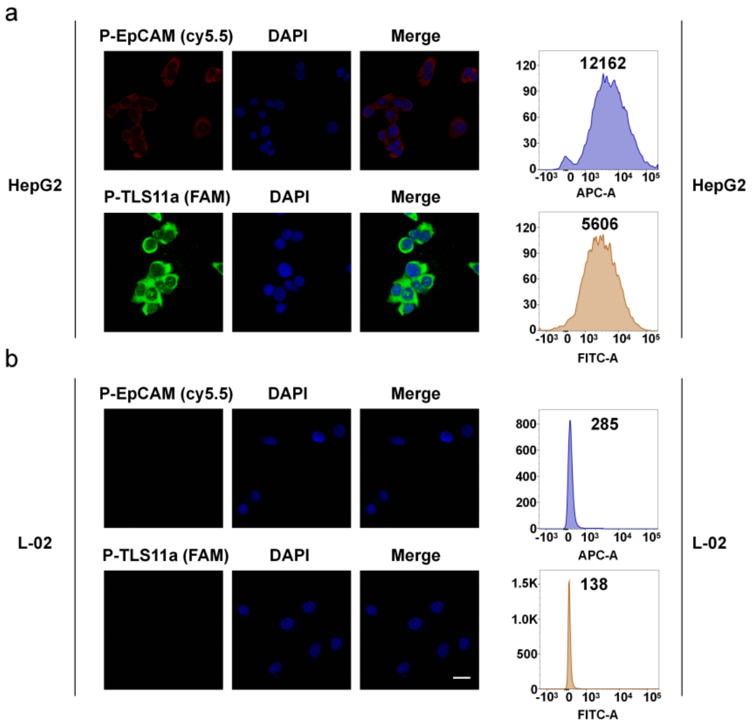

Figure S5. Confocal laser scanning microscopy (CLSM) and flow cytometry of receptor recognition probes EP (P-EpCAM, Cy5.5) and TP (P-TLS11a, FAM)

incubated with a) HepG2 cells and b) L-02 cells, respectively. Scale bar, 20 μm.

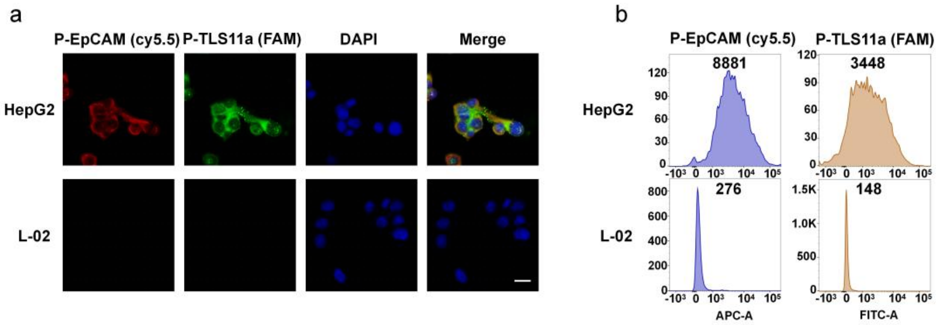

Figure S6. a) Confocal laser scanning microscopy (CLSM) and b) flow cytometry of receptor recognition probes EP (P-EpCAM, Cy5.5) and TP (P-TLS11a, FAM) incubated with HepG2 cells and L-02 cells, respectively. Scale bar, 20 μm.

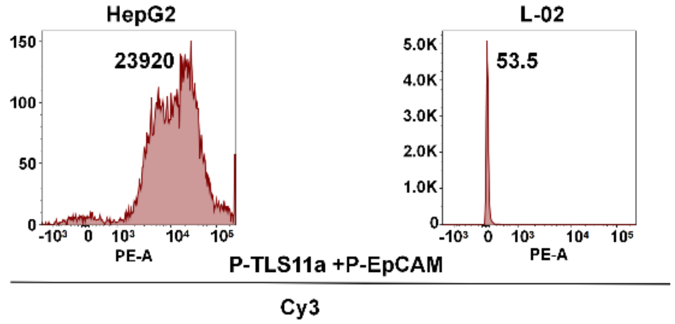

Figure S7. Flow cytometry of Panel-HCR incubated with HepG2 cells and L-02 cells for 60 min, respectively.

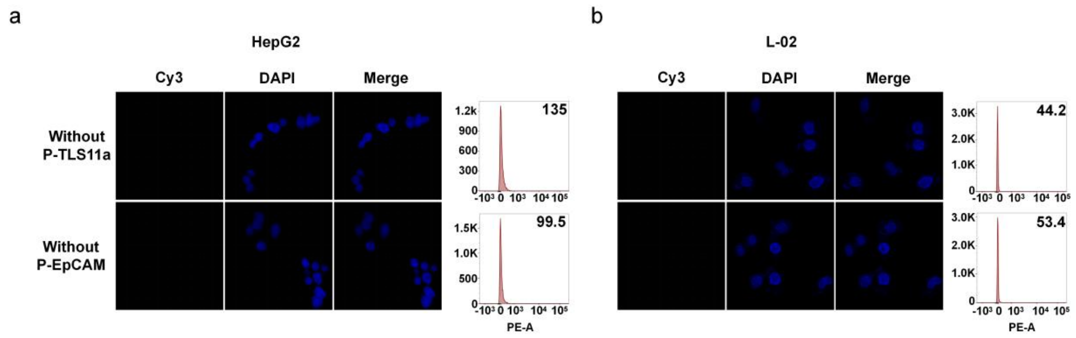

Figure S8. Confocal laser scanning microscopy (CLSM) and flow cytometry of a) HepG2 cells and b) L-02 cells without the receptor recognition probes TP (P-TLS11a) or EP (P-EpCAM). Scale bar, 20 μm.

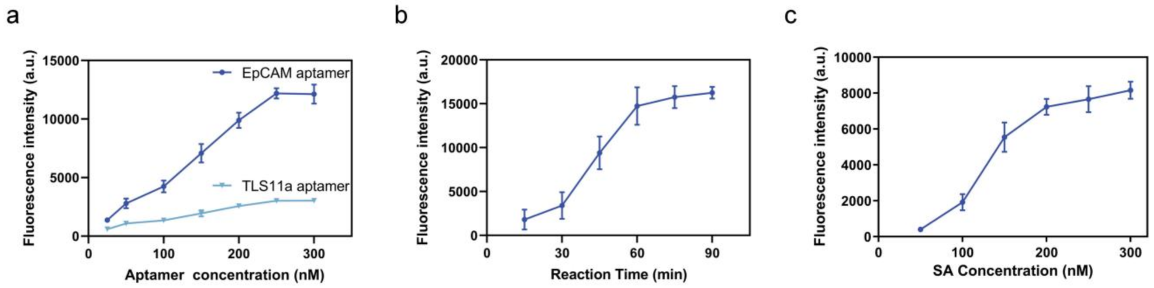

Figure S9. Optimization of a) receptor recognition probes concentration, b) reaction time and c) SA reaction concentrations (n=3), data are presented as mean ± SD.

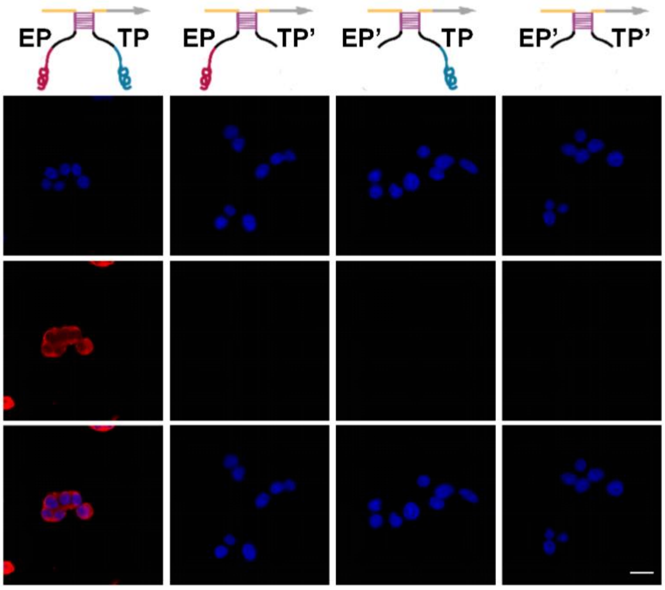

Figure S10. Receptor recognition probe integrity study. Scale bar, 20 μm.

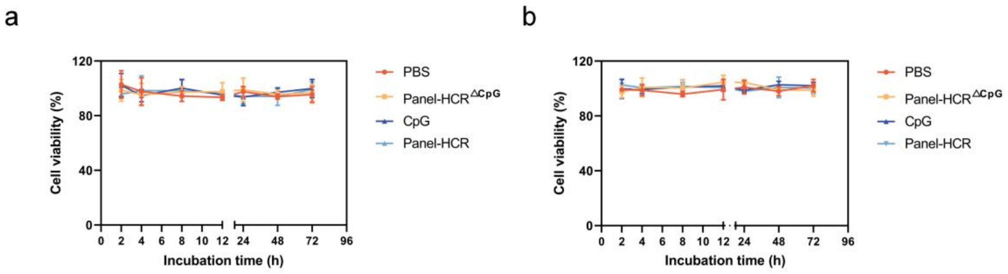

Figure S11. The CCK8 assay of (a Hepa1-6 cells and (b RAW264.7 cells with four treatments (n=3), data are presented as mean ± SD.

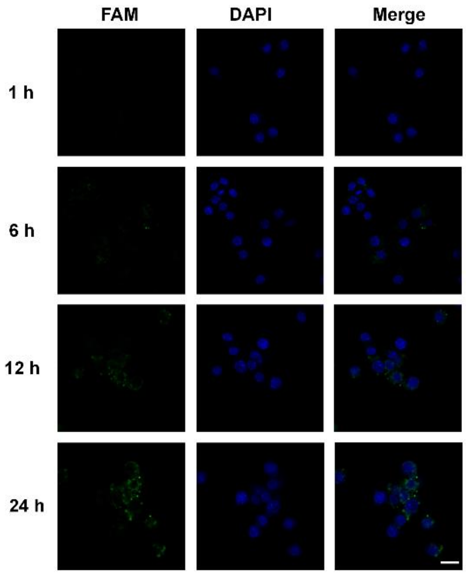

Figure S12. Confocal laser scanning microscopy image analysis of CpG enrichment in cells. Scale bar, 20 μm.

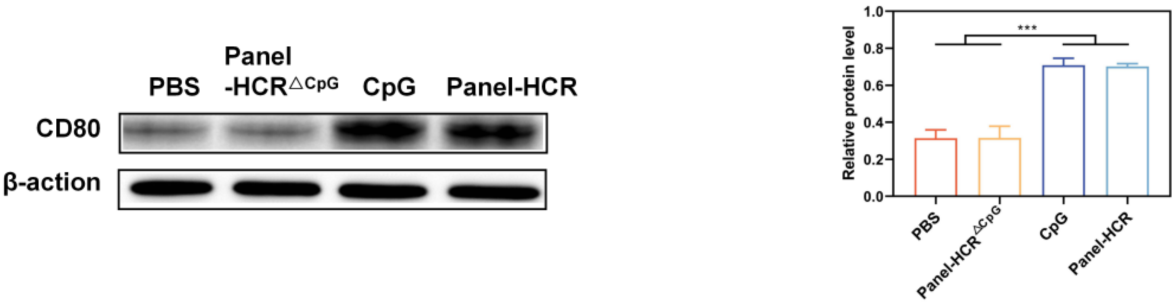

Figure S13. The CD80 (a marker of M1 TAMs) expression level of RAW264.7 cells with four treatments. ***$P < 0.001$ (n=3), data are presented as mean ± SD, and significance is determined using ANOVA with Post Hoc Multiple Comparisons (LSD).

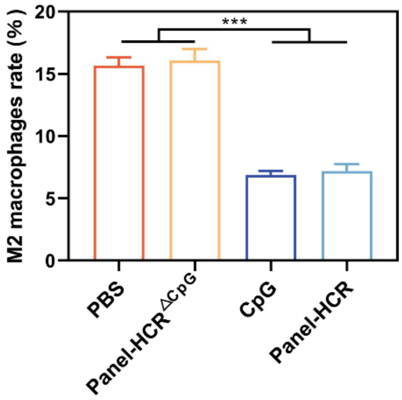

Figure S14. Statistical data of M2 TAMs rate with four treatments (n=3), data are presented as mean ± SD, and significance is determined using ANOVA with Post Hoc Multiple Comparisons (LSD). ***P < 0.001.

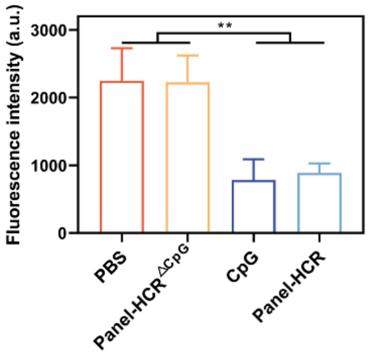

Figure S15. Statistical plot of the expression of the marker Arg-1 in RAW264.7 cells pretreated with IL-4 and then with four treatments (n=3), data are presented as mean ± SD, and significance is determined using ANOVA with Post Hoc Multiple Comparisons (LSD). **P< 0.01.

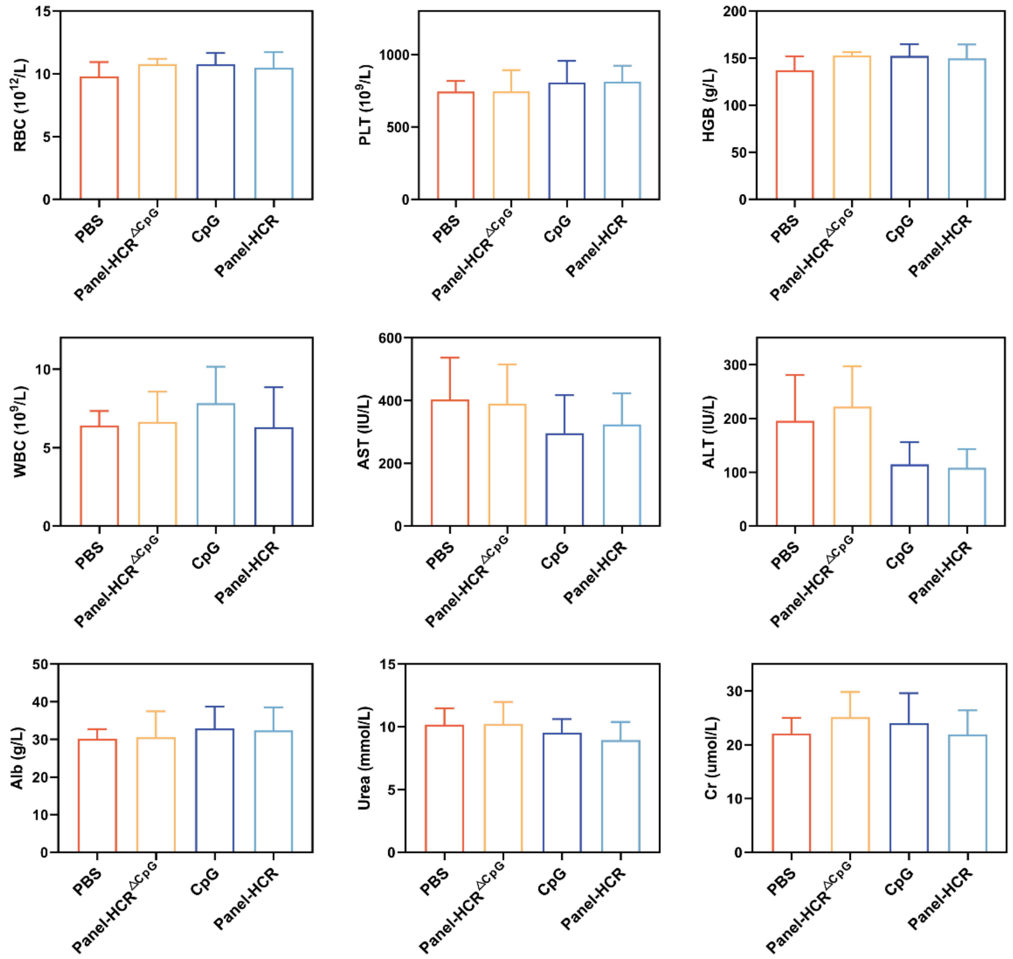

Figure S16. Hematological and biochemical indicators of tumor-bearing mice at the end of the treatment. Hematology indicators: red blood cell (RBC), platelet (PLT), hemoglobin (HGB), white blood cell (WBC). Liver function indicators: aspartate transaminase (AST), alanine transaminase (ALT), and albumin (Alb). Kidney function indicators: Urea and Cr (creatinine) (n=3), data are presented as mean ± SD.

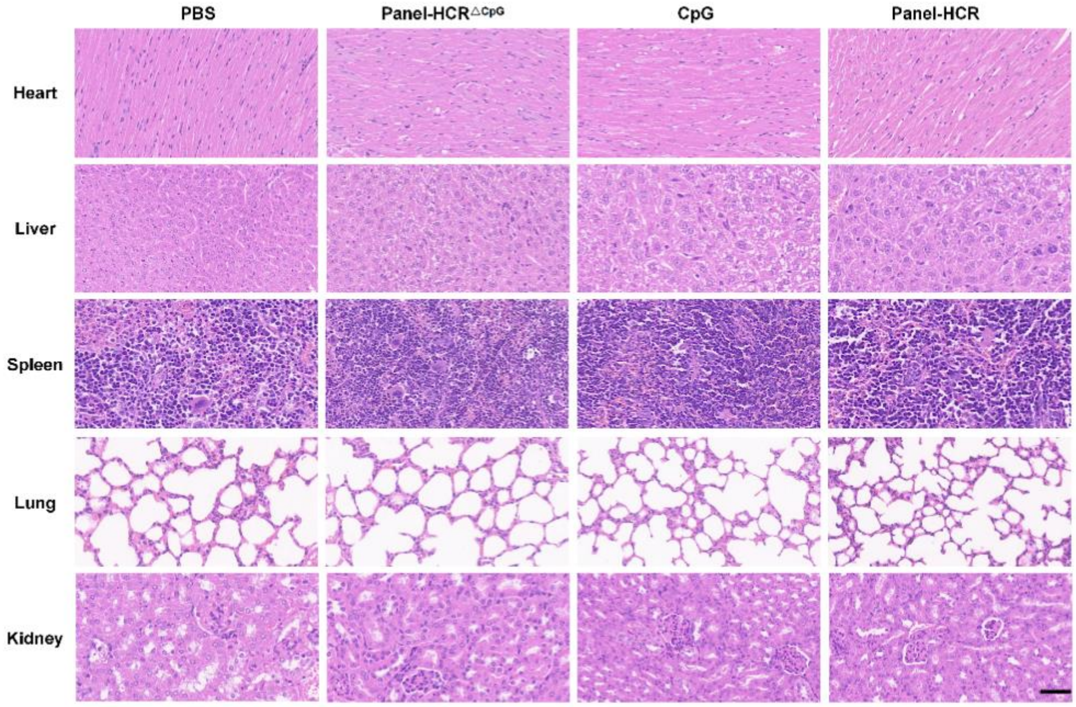

Figure S17. Histological examination of the main organs (hearts, livers, spleens, lungs and kidneys) of the tumor-bearing mice at the end of the treatment. Scale bar, 40 μm.

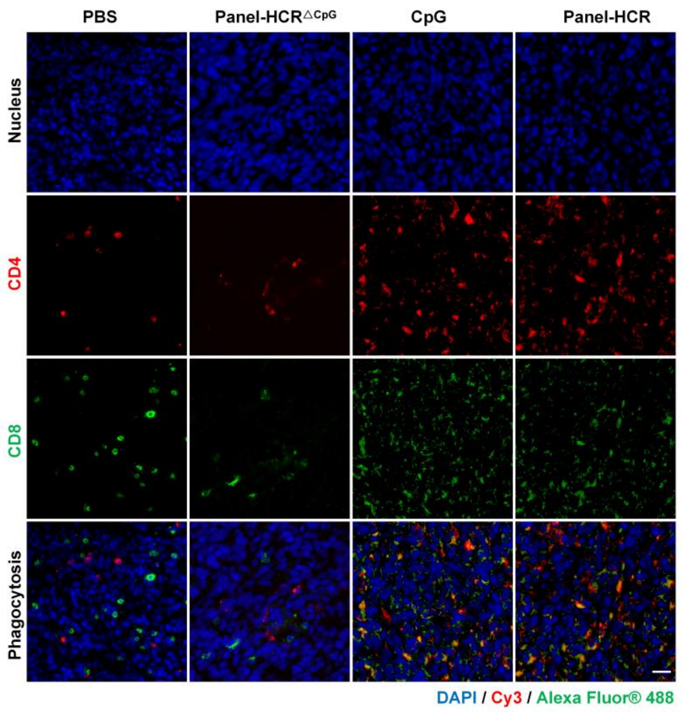

Figure S18. Immunofluorescence double staining analysis of $CD4^+$ T cells and $CD8^+$ T cells activation status in tumor tissue sections at the end of treatment. Scale bar, 20 μm.
